# Effects of vocoding and intelligibility on the cerebral response to speech

**DOI:** 10.1186/1471-2202-12-122

**Published:** 2011-11-30

**Authors:** Kuzma Strelnikov, Zoé Massida, Julien Rouger, Pascal Belin, Pascal Barone

**Affiliations:** 1 Université de Toulouse, Université Paul Sabatier, Centre de Recherche Cerveau et Cognition, 31062 Toulouse, France; 2 CNRS, UMR 5549. Hôpital Purpan, Toulouse, France; 3 CHU Purpan, Toulouse, France; 4 Centre for Cognitive NeuroImaging (CCNi) & Institute of Neuroscience and Psychology, University of Glasgow, Glasgow, UK; 5 Brain Innovation B.V., Maastricht, the Netherlands; 6 International Laboratories for Brain, Music and Sound, Université de Montréal & McGill University, Montreal, Quebec, Canada

**Keywords:** speech, voice, environmental sounds, vocoder, brain

## Abstract

**Background:**

Degrading speech through an electronic synthesis technique called vocoding has been shown to affect cerebral processing of speech in several cortical areas. However, it is not clear whether the effects of speech degradation by vocoding are related to acoustical degradation or by the associated loss in intelligibility. Using vocoding and a parametric variation of the number of frequency bands used for the encoding, we investigated the effects of the degradation of auditory spectral content on cerebral processing of intelligible speech (words), unintelligible speech (words in a foreign language), and complex environmental sounds.

**Results:**

Vocoding was found to decrease activity to a comparable degree for intelligible and unintelligible speech in most of the temporal lobe. Only the bilateral posterior temporal areas showed a significant interaction between vocoding and intelligibility, with a stronger vocoding-induced decrease in activity for intelligible speech. Comparisons to responses elicited by environmental sounds showed that portions of the temporal voice areas (TVA) retained their greater responses to voice even under adverse listening conditions. The recruitment of specific networks in temporal regions during exposure to degraded speech follows a radial and anterior-posterior topography compared to the networks recruited by exposure to speech that is not degraded.

**Conclusions:**

Different brain networks are involved in vocoded sound processing of intelligible speech, unintelligible speech, and non-vocal sounds. The greatest differences are between speech and environmental sounds, which could be related to the distinctive temporal structure of speech sounds.

## Background

In recent years, there has been an increasing number of studies that focused on the cerebral mechanisms of distorted speech processing [1-5]. These studies are warranted because distorted speech may constitute a model of complex speech comprehension, which is analogous to the complexity of imperfect real-life conditions. These studies should have clinical implications, because they serve to further elucidate the cerebral mechanisms of sound perception after cochlear implantations.

Modern cochlear implants (CI) allow deaf individuals to understand spoken speech and environmental sounds, and in some cases even to listen to music, although music perception usually remains poor [6-8]. However, auditory information delivered by the implant is spectrally degraded [9,10] and lacks some of the fine structure that is important for speech comprehension [11,12]. This technical limitation of CI contributes to a period of adjustment during the first months after implantation, when perceived sounds remain largely indecipherable and hearing is poor. In contrast, high levels of speech comprehension can be achieved over the first year of using the neuroprostheses [13,14].

As only a few (16-20 at present) electrodes are inserted along the cochlea during CI implantations, the frequencies delivered by each electrode are averaged across frequency bands, which reduces the number of frequencies available to the subject. To model the impact of this reduced number of frequency bands on the perceptual and neuro-physiological responses to sound in normal listeners, an artificial distortion called vocoding can be used. Vocoding averages frequencies in a varying number of frequency bands by applying special temporal envelopes (e.g., half-wave rectification and envelope smoothing), while preserving most of the slowly varying temporal cues. In other words, temporal and amplitude cues are preserved in each spectral band, but the spectral detail within each band is removed [9].

In normal subjects, voice discrimination and speech recognition with vocoded material improve as the number of vocoding channels is increased [9,15,16]. Variations of the number and spacing of frequency bands affects the brain in similar ways to changes in the number and placement of electrodes in CI. In a positron emission tomography (PET) study, Scott et al. [5] varied the number of vocoder channels between 1 to 16, thus increasing the level of intelligibility for the presented phrases. This vocoder-driven manipulation of intelligibility levels was found to correlate with bilateral brain activity in the superior temporal gyrus (STG) running lateral and anterior to the primary auditory cortex, with an additional peak in the left temporal pole. In a functional magnetic resonance imaging (fMRI) study of vocoded sentence perception, the number of vocoding channels was found to co-vary with cerebral activity in a large bilateral temporal cluster with peaks in the anterior superior temporal sulcus (STS), extending on the left side into the inferior frontal gyrus [4]. For isolated words, in contrast, the number of channels correlated with bilateral brain activity in the lateral STG, with a small bias to the right hemisphere [3].

These studies demonstrate a clear negative relationship along the bilateral STG between neuronal activity and the amount of vocoding-induced spectral degradation. These studies did not, however, dissociate the effects of vocoding-induced acoustical degradations and speech intelligibility decreases in these activity reductions, because they exclusively used stimuli composed of intelligible speech (IS).

Here, we attempt to dissociate the acoustical and intelligibility-related effects of vocoding on cerebral activity by comparing the effects of vocoding across intelligible speech (IS), unintelligible speech (US), and nonvocal environmental sounds (ES). The stimuli were presented to the subjects while they were performing a 1-back task either in their natural form or following degradation through vocoding with 2, 4, 8, or 16 channels. We expected that vocoding-induced acoustical degradation would affect processing of all of the stimuli in a similar manner. In contrast, we expected that vocoding-induced intelligibility losses would affect the processing of IS, but not of US or ES. Specifically, cerebral regions involved in intelligibility-related effects of vocoding were expected to show a significant Stimulus (IS, US) × Vocoding interaction.

A secondary goal of the present study was to ask whether the cerebral processing of different types of vocal information is differentially affected by vocoding, and whether the temporal voice areas (TVA) of auditory cortex preserve their sound-selectivity for voice after vocoding. Specifically, acoustical information that allows discriminations to be made between vocal and nonvocal sounds is expected to be preserved even at vocoding levels that significantly impair speech comprehension, suggesting possible dissociations at both the behavioural and cerebral levels [16]. To examine these issues, we compared the effects of vocoding on voice discrimination (voice/non-voice classification) and IS comprehension (IS/US classification) tasks performed outside the scanner, and its effects on the differential response to vocal and non-vocal sounds in the TVA. Our results demonstrate that different brain networks are involved in vocoded sound processing of IS, US and ES.

## Results

### Behavioural results

**Figure 1:**
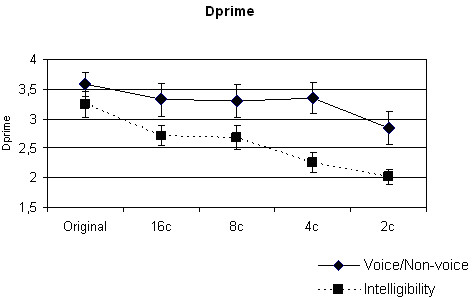
**Behavioral performance as a function of vocoder degradation**.

In both the Intelligibility and Voice Discrimination tasks, correct responses decreased with a decreasing number of vocoder channels (Figure 1). A two-way repeated-measures analysis of variance (ANOVA) with Task (Intelligibility, Voice Discrimination) and Degradation (no vocoding; vocoding using 16, 8, 4 or 2 channels) as factors showed a significant effect of Degradation on the performance of the subjects (F (4, 65) = 14.6, p < 0.001) (Figure 1). There was also a significant main effect of the type of Task F (1, 130) = 10.5, p < 0.01), and a significant interaction between Task and Degradation (F (4, 130) = 3.2, p < 0.05,). These data indicate that there was a greater vocoding-induced degradation of performance for the intelligibility task than for the Voice Discrimination task. Indeed, at intermediate levels of vocoding (4, 8 channels), voice versus non-voice discrimination performance remained close to normal levels whereas speech intelligibility was already markedly impaired (Figure 1).

### fMRI results

#### Effect of Category

**Figure 2:**
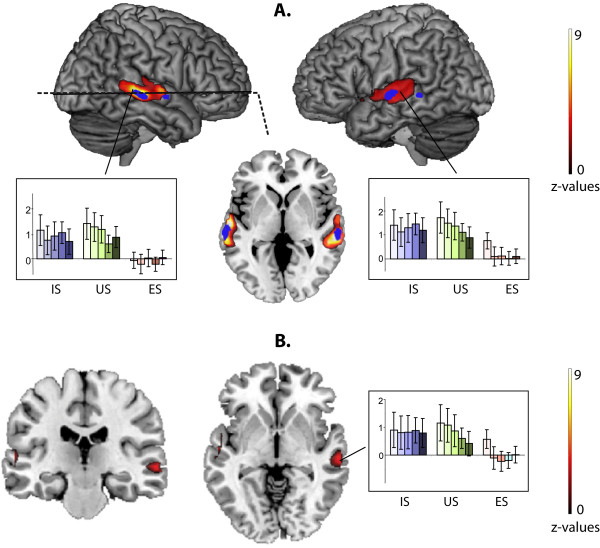
**Effect of stimulus Category**. A. Main effect of stimulus category. Vocoder degraded speech (degraded IS and US) vs. vocoder degraded E (IS+US vs. 2E). The horizontal slice is at the coordinate z = -3 (p < 0.0001). IS-**I**ntelligible **S**peech, US-**U**nintelligible **S**peech, ES-**E**nvironmental **S**ounds. In each group, the darker the color in the bar graphs of BOLD signal variation, the higher the level of vocoder degradation. In blue, areas from TVA localizer are presented at p < 0.05, FWE corrected. B. Degradation effect for speech vs. degradation effect for environmental sounds as defined by the expression (Speech_degraded_-Speech_normal_)-(Sounds_degraded_-Sounds_normal_). It can be algebraically rewritten as (Sounds_normal_-Sounds_degraded_)-(Speech_normal_-Speech_degraded_). The latter formula makes clear that the difference between normal and degraded stimuli in the right STS is smaller for speech compared with non-speech sounds. In each group, the darker the color in the bar graphs of BOLD signal variation, the higher the level of vocoder degradation.

A significant main effect of Category in our 3×5 full factorial design was observed in the bilateral STS/STG (Figure 2A, Table 1).

**Table 1:** Main effect of category, regression of BOLD signal variations with the levels of vocoder degradation and its interaction between IS and US.

Brain region	N voxels	p corr	z-value	x	y	z
**Main effect of category**						
L STG	93	0.000	Inf	-60	-21	-3
R STS	104	0.001	Inf	60	-24	-3
**Regression with the levels of vocoder degradation (logarithmic)**						
L STG/STS	987	0.000	Inf	-57	-15	6
		0.000	Inf	-48	-24	6
		0.000	Inf	-51	-6	-3
R STG/STS	495	0.000	Inf	57	-24	-3
		0.000	Inf	57	-27	6
		0.000	Inf	55	-27	15
R cerebellum	532	0.000	Inf	18	-75	-18
		0.000	7.72	18	-66	-21
		0.000	7.63	33	-45	-30
L cerebellum	42	0.000	7.17	-18	-72	-39
		0.003	5.21	-9	-75	-33
R putamen	17	0.000	6.05	24	9	0
		0.028	4.72	24	3	-9
**Interaction of vocoder degradation effect between IS and US**						
L STG, post.	31	0.002*	2.84	-57	-18	15
R STG, post.	33	0.002*	2.89	57	-27	15
R insula	18	0.011*	2.29	42	-15	-9
L lateral sulcus, post	10	0.02*	2.05	-42	-33	9
R cerebellum	19	0.003*	2.74	21	-36	-30

Cluster size is indicated at the level of p = 0.05, FWE-corrected, p-values are indicated at voxel level. STS-superior temporal sulcus.

* uncorrected p-values from region of interest analysis using as regions of interest those of the "All effect of interest" contrast.

As 80% of our stimuli were degraded stimuli, we determined whether the Category effect was related to the differences between processing of degraded speech and degraded environmental sounds. We collapsed the beta values across the degrees of vocoding and compared brain activity in response to vocal sounds and to environmental sounds (IS+US vs. ES). The peaks were the same as those observed in the Category effect analysis (Table 2 Figure 2A). The same analysis (IS+US vs. ES) did not provide any significant results for the non-degraded stimuli, likely because of their small number in the paradigm. Planned comparisons revealed no difference between activity induced by IS versus US (p > 0.5, uncorrected). In other words, the greater responses to speech in these regions were not related to intelligibility.

**Table 2:** Comparison of speech and non-speech.

Brain region	N voxels	p corr	z-value	x	y	z
**Vocoded speech (IS+US) > vocoded E**						
L STG	86	0.000	6.24	-60	-21	-3
R STS	99	0.000	6.08	57	-24	-3
**Degradation effect for speech > degradation effect for E**						
R STS	43	0.005	5.11	57	-24	-6

By "speech" here, we mean the pooled effect of intelligible speech (IS) and unintelligible speech (US). Cluster size is indicated at the level of p = 0.0001, uncorrected. FWE-corrected p-values are indicated at voxel level, except for those significant only at cluster level, marked as (clust). STS-superior temporal sulcus, STG-superior temporal gyrus.

### Effect of Degradation

**Figure 3:**
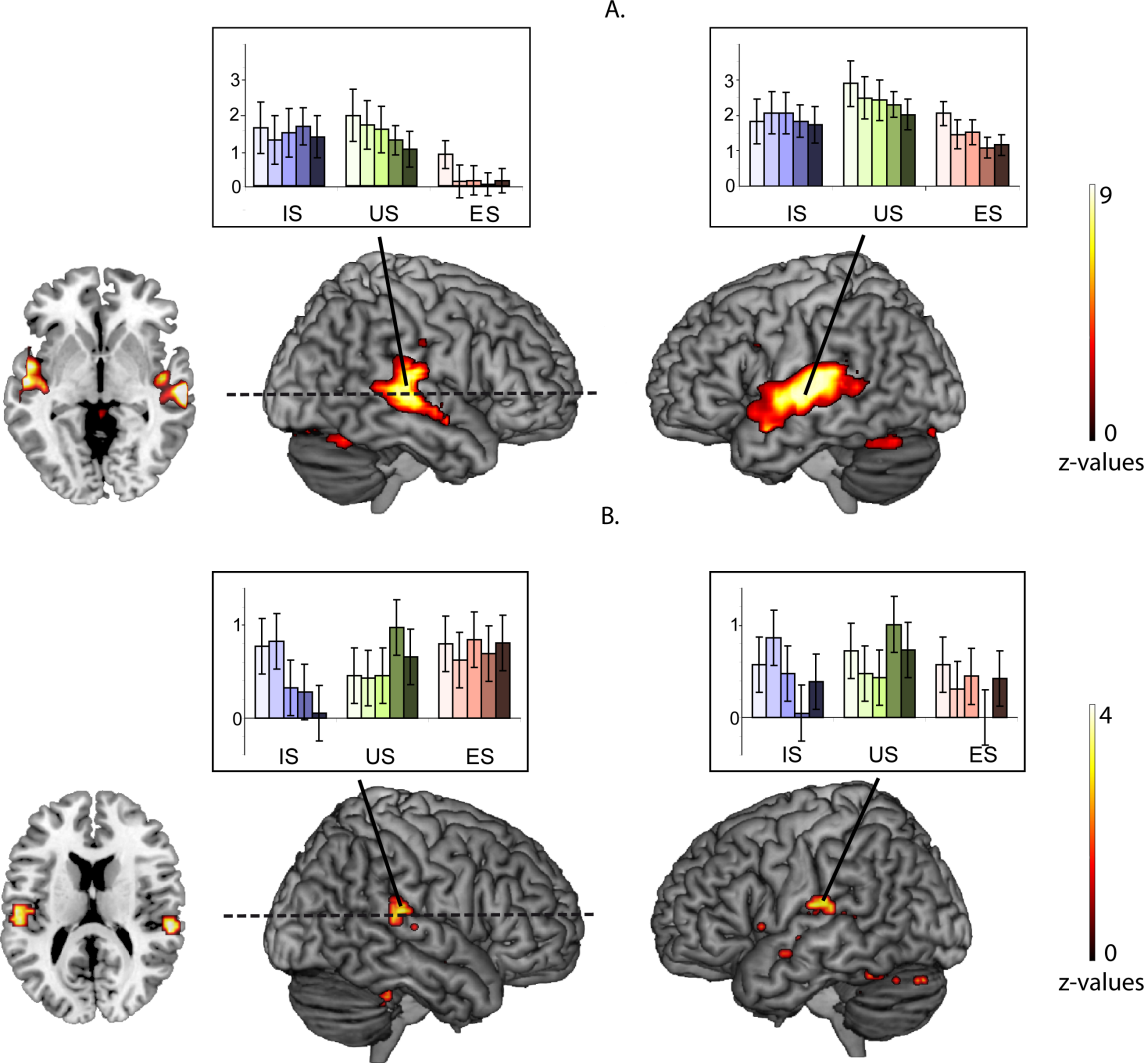
**Effect of vocoder degradation on BOLD signal variation**. A. General regression with vocoder degradation. Plots are presented for the peaks in the left and right temporal regions: L STG/STS at (-57 -15 6) and R STG/STS at (60 -24 -3), the horizontal slice at the coordinate z = -3 (p < 0.0001). IS-**I**ntelligible **S**peech, US-**U**nintelligible **S**peech, ES-**E**nvironmental **S**ounds. In each group, the darker the color, the higher the level of vocoder degradation. The level of degradation in this analysis was transformed to the logarithmic scale being the logarithm of the inverse number of channels n: log(1/n). In each group, the darker the color in the bar graphs of BOLD signal variation, the higher the level of vocoder degradation. B. Interaction between Category and Degradation (factorial analysis of IS, US and ES, contrast weights restricted to IS and US) in the regions of interest from the "All effects" contrast. Plots are presented for the peaks in the left and right temporal regions: L STG at (-57-18 15) and R STG at (57-27 15), the horizontal slice at the coordinate z = 15. In each group, the darker the color in the bar graphs of BOLD signal variation, the higher the level of vocoder degradation.

The linear approach used in the 3 × 5 full-factorial design did not detect brain areas sensitive to vocoder degradation (p > 0.5). Therefore, using regression analysis, we determined the relationship between the logarithmic values of the number of channels (using a value of 64 channels for the natural stimuli) and brain activity. Using this model, we found a significant correlation between BOLD signals and the level of vocoder degradation in a large bilateral area of the STG/STS and in the cerebellum (Figure 3, Table 1).

### Category × degradation interaction

No region showed a significant Category × Degradation interaction in the full factorial design, indicating similar effects of vocoder degradation on IS and US. As we had an *a priori *hypothesis that there would be a statistical interaction between degradation and intelligibility, we conducted a hypothesis-driven analysis of these interactions in a restricted number of regions that are involved in general speech and sound processing based on the "All effects of interest" contrast (used as a mask at p = 1e-07). A differential influence of vocoder degradation on IS, US, and ES was found bilaterally in the lateral superior temporal cortex and in the right cerebellum (Table 1 Figure 3B). In these regions, degradation-related deactivations were more pronounced for IS than for US or ES.

### Mean degradation effect

As an alternative way to examine the effects of vocoder degradation, we compared the differences in brain activity between the degraded and natural stimuli. To examine these "degraded vs. normal" differences, we pooled the effects of the degradation levels for each stimulus category. When comparing the differences between "degraded vs. natural" stimuli, we found that the reduction in activity in the right STS caused by vocoder degradation was smaller for IS and US than for ES (Table 2 Figure 2B). This result suggests that the TVA remains specifically sensitive to speech compared with non-vocal sounds, even when the stimuli are degraded. Though no significant interaction was found in the whole brain analysis between the main factors of Category and Degradation, collapsing across the levels of degradation revealed a group specific effect. Notably, no difference in the similar "degraded vs. natural" analysis was found between IS and US (p > 0.3, uncorrected).

### Region of interest approach

We used brain regions issued from the TVA localizer and applied them to the IS and US images from the event-related part of the study. These functionally defined ROIs included the TVA along the bilateral STS and amygdala (Table 3). Using both linear and logarithmic approaches, no correlation with the vocoder degradation levels was found in the TVA areas (p > 0.1), suggesting that they were comparably responsive to normal and degraded speech.

**Table 3:** Brain regions from the comparison of voice vs.environmental sounds in "TVA localizer".

Brain region	N voxels	p corr	z-value	x	y	z
R STS middle	259	0.000	Inf	60	-30	0
R STS anterior	49	0.004	5.24	54	12	-21
L STS	113	0.000	6.07	-57	-30	-3
L STS	65	0.000	5.98	-57	-3	-12
R amygdale	55	0.002	5.38	21	-6	-15
L amygdale	31	0.000	5.84	-21	-6	-15

Cluster size is indicated at the level of p = 0.0001, uncorrected. FWE-corrected p-values are indicated at voxel level. STS-superior temporal sulcus, STG-superior temporal gyrus.

Comparing activations from the TVA localizer and from the main effect of Category in the bilateral STS/STG (Table 1), we found that the Euclidian peak-to-peak distance between clusters is 9.5 mm on the left and 6.7 mm on the right, which is greater than the 6 mm Gaussian kernel used for smoothing. However, at the same threshold (p < 0.05, family-wise error (FWE) corrected), 47% of the clusters on the right and 58% of the clusters on the left from the main Category effect overlapped with clusters from the TVA localizer (voice vs. environmental sounds).

Comparing activations from the TVA localizer with those from the regression with the levels of vocoder degradation (Table 1) in the bilateral STS/STG, we found that the Euclidian peak-to-peak distance between clusters is 17.5 mm on the left and 7.3 mm on the right, which is greater than the 6 mm Gaussian kernel used for smoothing. At the same threshold (p < 0.05, FWE corrected), 16% of the clusters on the right and 3% of the clusters on the left from the regression analysis overlapped with clusters from the TVA localizer.

Thus, based on the TVA localizer, the areas for normal voice processing are spatially distinguishable from the areas correlated with vocoder degradation of speech. Specifically, for the Category effect, there is approximately 50% overlap between TVA localizer and the degraded stimuli.

## Discussion

We have used vocoded IS, US, and ES with different levels of vocoder degradation to differentiate the modulation of cortical activity by acoustical degradation and a reduced speech intelligibility. Comparisons with environmental sounds suggest that certain brain areas retain their speech specificity even when the presented stimuli are degraded, probably due to the important temporal structure of speech sounds [9,10,12]. We have been able to delineate a bilateral region in the posterior STG a region that plays a different role in processing vocoder degraded IS and US, suggesting that this region may be implicated in the increased computational efforts required to process linguistic information in difficult acoustical situations.

### Vocoder degradation and speech processing

In the present study, the bilateral STG activations related to vocoder degradation confirm earlier findings for vocoder degraded words [3,5]. These STG activations are different for speech compared to ES. In a large portion of the STG, vocoder degradation effects are probably related to speech-specific acoustic properties [9,10,12,17], which are present in IS and US. Obleser et. al. [3] reported the presence of areas where there is covariation between vocoder degradation and BOLD activity in bilateral STG/STS regions, which overlap with areas found in the present study. As they used only words for stimulation, they could not clarify whether their observed effects were specific to the lexico-semantic level of processing or whether it reflected increased demand of the complex acoustical analysis under the degraded acoustic conditions. Though there is no interaction between IS and US in our whole brain approach, significant interactions do exist in the bilateral posterior temporal areas and the cerebellum using regions of interest from the "All effects of interest" contrast (Table 1 Figure 3B). This temporal region, located outside the areas found by Obleser et al. (2008), shows a more marked degradation-related deactivation for IS than for unintelligible stimuli (US and ES) (Figure 3B). Such differential effects of IS and US in the posterior STG suggests that these regions are involved in processing vocoder degradation of US and ES. The emergence of the additional neuronal support for the unintelligible sounds (US and ES) in adverse conditions is consistent with our findings that the negative effects of vocoder degradation on task performance were more marked for the linguistic Intelligibility task (US/IS) than for the Voice/Non-voice Discrimination task (Figure 1).

As mentioned in the Introduction, vocoder degradation is analogous to imperfect real-life conditions. As such, understanding the functional role of the speech degradation-related network may lie in the perception of ecological noisy speech. Indeed, the study of word perception with babbling noises from multiple talkers [18] revealed that the same areas in the bilateral, posterior STG were more responsive to speech contained in noise than for isolated speech. However, this study did not demonstrate whether this effect was specific for speech intelligibility or was a more general effect of differences between speech and non-speech sounds. Interestingly, they reported increased activity in this area in response to words in noise, which is contrary to the decreased response to vocoded words in the present study. This may indicate that the reported area is involved in extracting spectral information from speech in adverse conditions, which is preserved in noisy speech but is degraded by vocoding. The level of deciphering of this spectral information from vocoded sounds is sufficient for speech and sound recognition, but is insufficient for speech intelligibility. Following vocoder degradation, poorer recognition of speech intelligibility compared with voice/non-voice recognition was also confirmed by our behavioural data (Figure 1).

The results of the present study suggest that the correlations with vocoder degradation reported in previous studies with only linguistic stimuli [3-5] cannot be attributed to linguistic processes, and areas in the temporal cortex and in the cerebellum are involved in speech intelligibility. Moreover, we found bilateral effects of vocoder degradation on activity in the cerebellar hemispheres, a result not described in previous studies of vocoded speech processing [3,5]. The cerebellum is involved in speech processing [19], especially in difficult acoustical conditions such as noisy environments [18] and in patients with CI [20].

### Vocoder degradation and temporal voice areas (TVA)

The analysis of the Main category effects for the degraded stimuli revealed cortical regions that exhibit approximately 50% overlap with voice-specific regions using more ecologically-relevant voice stimuli from the TVA localizer. The locations of the peaks for the degraded speech stimuli were displaced compared to the peaks for the voice stimuli obtained from the voice localizer analysis. In general, there is only a weak and non-significant effect of speech degradation on peak activity in the TVA. However, because of overlap between the TVA and areas that are sensitive to vocoder degradation, the peripheral regions of the TVA showed greater involvement in the processing of degraded speech.

The spread of activity for degraded speech is radial from the normal TVA peaks with the most pronounced directions along the anterior-posterior axis. We did not observe any effects of vocoder degradation at the peaks of the voice specific areas issued from the TVA-localizer. Moreover, these voice specific areas were spatially distinguishable from those related to vocoder degradation. These results suggest that activity at the peaks of these TVA areas is not influenced by the high levels of vocoder degradation. A plausible explanation for this possibility is that peripheral activity in TVA-related networks deciphers voice information and transmits it to the regions involved in normal voice analysis.

In addition, global degradation effects were different between speech and ES in the right STS. Thus, this analysis revealed areas that are resistant to degradation of speech in comparison with degradation of ES. It is possible that speech survives degradation better than non-speech sounds because the temporal cues contained in speech are relatively preserved following vocoder degradation (vocoding). In vocoding, a process that mimics the effects of a cochlear implant processor, global temporal information, i.e. the envelope, is preserved, while the fine spectral structure is degraded [9]. Such differential effects makes speech less sensitive to degradation than non-speech sounds (see [16]). Whether intelligible or not, speech has a specific temporal structure [17], which makes it distinguishable from ES. It has been demonstrated that modulating broadband noise with temporal envelopes extracted from speech does not prevent a subject from using only temporal cues to recognize a sound as speech [9]. Therefore, the distinction between speech and non-speech sounds can be made based exclusively on temporal features. Retention of speech recognition at high levels of vocoder degradation may be explained by the preservation of speech-specific temporal structure in the speech stimuli.

In a study of degraded voice perceptions [21], the scrambled voices had the same amplitude values as those in the original signal (hence preserving the overall energy) but at different frequencies, which made these sounds totally unrecognizable as voices. Speech sounds elicited greater responses than their scrambled version in nearly all parts of auditory cortex including primary auditory cortex. In contrast, non-speech vocal sounds elicited greater responses than their scrambled version only in the middle and anterior portions of the right STS. Thus, it was suggested that the scrambling degradation technique has a relatively small effect on the activity of voice-specific neural networks compared to its effect on speech-specific networks. This result is in line with our presently presented data, where vocoder degradation had a small effect on speech-specific activity and on discrimination scores, but had a larger effect on speech intelligibility as reflected by brain activity in the posterior STG and discrimination scores.

## Conclusions

The brain networks involved in vocoded sound processing are different when the stimuli are composed of IS, US, or ES. The recruitment of speech-specific networks in the temporal regions when degraded speech is presented follows a radial and anterior-posterior topography with regard to normal voice responses. This may be related to the processing of speech-specific temporal cues, which are relatively preserved by vocoder degradation.

## Methods

### Subjects

Fifteen English-speakers with normal hearing, 9 females (mean age 26, range 19-41) participated in the study. No participant reported having any auditory or neurological diseases, and they all had normal or corrected to normal vision. All of the subjects gave their full and informed consent prior to their participation in accordance with the Declaration of Helsinki (1968). The study was approved by the local research ethics committee of Glasgow University.

### Stimuli

We used a 3 × 5 (Category × Degradation) full factorial design, with the stimulus categories IS (26 frequent English words, with half spoken by a female voice), US (26 unfamiliar words in Finnish and Japanese, with half spoken by a female voice) and non-vocal ES (26 industrial sounds, non-living nature sounds, or bird and animal sounds). The vocoding levels were 2, 4, 8, and 16 channels and no vocoding (unchanged, original sounds). Each stimulus was presented in its original form plus 4 vocoder-degraded forms resulting in 26*5 = 130 presented items per category, for a total of 390 stimuli. The durations of the stimuli were (mean ± standard deviation) IS: 740 ± 20 msec, US: 730 ± 100 msec, and ES: 740 ± 20 msec (no significant difference, p < 0.87, F(2, 75) = 0.14). The sound intensity was normalized for each stimulus by RMS power.

#### Behavioural testing

The behavioural part of the study was conducted in an fMRI simulator after the scanning session. The fMRI simulator reproduced the environment of the fMRI scanner, including the continuous scanning noises. In two tasks in a counterbalanced order, the subjects were presented the same stimuli with the same levels of degradation as in the scanner. In the Intelligibility task, subjects were asked to distinguish between IS and US, i.e., to decide whether the heard voice was speaking an intelligible English word or not. In the Voice Discrimination task, subjects were presented with US and ES stimuli and asked to decide whether the sound was a voice or not. In both tasks, the stimuli were presented in a pseudo-random order, and the subjects indicated their responses using a computer mouse.

#### fMRI scanning

In the main functional run, stimuli corresponding to the 3 categories × 5 degradation levels were presented in a pseudo-random order with 20% of the presented stimuli consisting of randomly occurring null-events. For each stimulus category, 26 (13 male and 13 female voice) stimuli were randomly played (ISI = 4 sec, stimuli duration 0.7 ± 0.1 (SD) sec). The subjects were instructed to press a button when they heard a repetition (1-back task). There were no other repetitions in the stimuli presentation. Except for this "orthogonal" task, there were no other stimuli repetitions.

In the "TVA localizer" [22] part of the study, stimulus blocks of 8 sec were presented in an efficiency-optimized order, consisting of human vocalizations (speech and non-speech) or non-vocal ES (the stimuli are available at http://vnl.psy.gla.ac.uk/resources_main.php), intermixed in 33% of the noiseless blocks. The subjects were instructed to listen attentively and passively.

For both fMRI scans, the auditory stimuli were presented binaurally through pneumatic headphones, sealed by foam ear inserts, and further shielded by plastic ear defenders (providing an attenuation of fMRI scanning noise of about 30 dB), with a sound-pressure level of 85-90 dB.

### Imaging details

Scanning was performed in a 3T MRI system (Siemens) at the Neuroimaging centre of Glasgow University. Functional scans were acquired with a single-shot echo planar gradient-echo (EPI) pulse sequence (TR = 2 s, TP = 30 ms, flip angle = 77°, FOV = 215 mm, matrix = 64 × 64). The 32 axial slices (resolution 3.75 × 3.75 mm in-plane, 5-mm thickness) in each volume were aligned to the AC-PC line, covering the whole brain. A total of 498 volumes per subject were acquired after T1 saturation for the event-related part of the study and 310 volumes for the "TVA localizer" part. Scanner noise was continuous throughout the experiment. After the functional scans, T1-weighted anatomical images were obtained for each participant (1 × 1 × 1 mm resolution).

### Image processing and analysis

Image processing and statistical analysis were performed using SPM5. The imaging time series was realigned to the first volume to correct for interscan movement. To account for differences in sampling time of different slices, the voxel time series were interpolated using 4th degree b-spline interpolation and re-sampled using the 1st slice at the anterior-posterior commissural line as the reference. A T1-weighted anatomical MRI (1 mm×1 mm×1.5 mm voxel slice) was obtained for each subject, co-registered with the mean realigned functional image and normalised using the parameters determined for the functional images. A mean anatomical image was created from the subjects' individual scans, onto which activations were overlaid for anatomical localisation. Finally, the functional images were spatially normalised to a standard MNI space to allow for group analysis. Functional data were smoothed using a 6 mm full-width at half maximum (FWHM) isotropic Gaussian kernel. The data were analysed by modelling the evoked hemodynamic responses with canonical hemodynamic response functions for the fixed-effects general lineal model per subject.

In the event-related part of the study, 15 contrasts per subject were created. For each of the 3 categories of stimuli, 5 contrasts were created (4 levels of vocoder degradation and the natural sound relative to the baseline). These contrasts were used at the group-level in a random-effect analysis using a full 3 × 5 (Category × Degradation) factorial design. The "All effects of interest" contrast (used as a mask at p = 1e-07) served for the hypothesis-driven analysis of IS and US interaction in the flexible factorial design with IS, US and ES stimuli; contrast weights were restricted to IS and US stimuli. In the TVA -localizer part of the study, for each subject, the voice and non-voice contrasts were created. For the 2^nd ^level analysis, these contrasts were compared for the group using a one-sample t-test. Brain areas showing significant activity differences in the "voice vs. non-voice" contrasts (the TVA) were used as regions of interest in the full factorial analysis of the event-related design using the MarsBaR toolbox.

The FWE corrected value of p < 0.05 was used for the whole-brain analysis in both parts of the study.

## Authors' contributions

KS planned the experiment, conducted the study, analysed the fMRI data of vocoder degradation and wrote the article. ZM analysed the behavioural data and fMRI data of the voice localizer. JR prepared the stimulation programs and conducted the study. P. Belin planned the experiment, analysed the fMRI data of vocoder degradation, and wrote the article. P. Barone planned the experiment and wrote the article. All authors read and approved the final manuscript.
